# Systematic review: the use of tacrolimus in steroid-refractory ulcerative colitis

**DOI:** 10.1177/20406223251333570

**Published:** 2025-06-12

**Authors:** Vinay Jangra, Dilsan Yilmaz, Delvene Soares, Fiona Taylor, Stefano Andreani, Alexander Hotouras

**Affiliations:** Department of Colorectal Surgery, Barts Health NHS Trust, Whipps Cross Hospital, Whipps Cross Road, London E11 1NR, UK; Queen Mary’s University, London, UK; Department of Colorectal Surgery, Barts Health NHS Trust, London, UK; Department of Colorectal Surgery, Barts Health NHS Trust, London, UK; Department of Colorectal Surgery, Barts Health NHS Trust, London, UK; Department of Colorectal Surgery, Barts Health NHS Trust, London, UK; Department of Colorectal Surgery, Barts Health NHS Trust, London, UK

**Keywords:** calcineurin inhibitors, immunosuppressants, steroid-refractory, tacrolimus, ulcerative colitis

## Abstract

**Background:**

Steroid-refractory ulcerative colitis (SR-UC) is a severe form of ulcerative colitis (UC) that persists despite high-dose corticosteroid therapy. Management is challenging and often requires biologics, immunosuppressants or colectomy. Tacrolimus, a calcineurin inhibitor with immunomodulatory effects, has emerged as a potential alternative, though its efficacy, safety and long-term outcomes remain uncertain.

**Objectives:**

This systematic review evaluates the efficacy and safety of tacrolimus in SR-UC, compares it with anti-tumour necrosis factor (TNF) agents, and examines its role in patients with steroid-refractory ulcerative proctitis (SR-UP).

**Design:**

A systematic review was conducted following Preferred Reporting Items for Systematic Reviews and Meta-Analyses guidelines. Studies were assessed using the Oxford Centre for Evidence-Based Medicine framework.

**Data sources and methods:**

A comprehensive search of PubMed and Medline (1998–2025) identified studies reporting clinical remission, response rates, adverse effects and colectomy rates. Subgroup analyses compared oral tacrolimus with anti-TNF therapy and evaluated topical tacrolimus in SR-UP.

**Results:**

Seventeen studies (658 patients) met the inclusion criteria. Tacrolimus demonstrated variable efficacy, with clinical remission rates of 9.4%–75.6% and response rates of 28.6%–89.6%. Adverse events, mainly neurotoxicity and nephrotoxicity, occurred in 16.7%–53.0% of cases, sometimes leading to treatment discontinuation. Colectomy rates ranged from 6.1% to 62%. No significant difference was observed between tacrolimus and anti-TNF agents in remission induction, though anti-TNFs had superior long-term outcomes. Topical tacrolimus showed promising efficacy in SR-UP (73.0%–75.0% remission) but was associated with high adverse event rates (45.0%–67.0%).

**Conclusion:**

Tacrolimus is a viable option for SR-UC, particularly in combination therapy, but its long-term efficacy remains uncertain. While it is comparable to anti-TNFs for short-term remission, high colectomy rates and adverse effects require careful monitoring. Topical tacrolimus shows promise for SR-UP but requires standardised dosing. Further large-scale trials are needed to optimise treatment strategies and evaluate long-term safety.

**Trial registration:**

The review has been registered with PROSPERO (CRD42023432827), the international prospective register of systematic reviews.

## Introduction

Ulcerative colitis (UC) is a chronic inflammatory bowel disease (IBD) characterised by persistent inflammation and ulceration of the colon and rectum, leading to symptoms such as diarrhoea, abdominal pain and rectal bleeding. The management of UC requires a multidisciplinary approach, with an overall goal to induce and maintain remission, achieve mucosal healing, prevent complications and the ultimate goal of improving a patient’s quality of life.^1^

A subset of UC, steroid-refractory ulcerative colitis (SR-UC), is defined by persistent disease activity despite 4 weeks of treatment with prednisolone at 1 mg/kg/day.^2,3^ This condition often necessitates more aggressive management strategies to control symptoms and achieve remission, including the use of biologic therapies such as infliximab and vedolizumab, along with immunosuppressants such as cyclosporine and methotrexate.^4^ The persistent nature of SR-UC, along with the limited efficacy and side effects of current therapies, severely impacts patients’ quality of life and complicates treatment decisions. This underscores the critical need for novel therapeutic approaches to improve patient outcomes.

Building on the success of cyclosporine as salvage therapy,^5^ tacrolimus, a potent immunomodulatory agent and calcineurin inhibitor, has emerged as a promising alternative for patients with SR-UC.^6^ Tacrolimus exerts its immunosuppressive effects by binding to FK506-binding proteins, leading to the inhibition of phosphatase activity and suppression of interleukin (IL)-2 transcription. This process occurs through impaired nuclear factor of activated T-cell translocation, which regulates IL-2 expression and T-cell activation.^7^ Tacrolimus has shown effectiveness in preventing graft-versus-host disease and organ rejection following transplants^8^ as well as in treating autoimmune conditions such as autoimmune enteropathy.^9^ These successes suggest its potential in managing the chronic inflammation characteristic of SR-UC.

Systemic use of tacrolimus may be associated with side effects, leading to interest in topical delivery as a potential alternative.^10^ Topical administration can provide localised immunosuppressive effects, minimising systemic exposure and reducing adverse events.^11^ It is particularly effective for treating distal colonic disease, targeting inflammation directly while limiting systemic drug exposure.^12^ However, topical therapies are often underused due to patient preferences and healthcare provider unfamiliarity.

Further investigation is needed to fully assess tacrolimus’ safety, efficacy and role in the treatment of SR-UC. This systematic review critically evaluated the current evidence on both systemic and localised tacrolimus, which aimed to determine its potential as a viable therapeutic option for patients with limited alternatives. Doing so will contribute to valuable insights into the clinical research landscape and may help inform future treatment decisions for this challenging condition.

## Methods

### Search strategy

A systematic search was conducted on the PubMed and Medline databases from 1998 to 2025 to comprehensively identify literature pertaining to the utilisation of tacrolimus in the context of SR-UC. The search strategy was devised to encompass relevant MeSH terms, including ‘tacrolimus’, ‘steroid’, ‘refractory’ and ‘ulcerative colitis’.

### Inclusion and exclusion criteria

Included in this systematic review are studies specifically evaluating the efficacy of tacrolimus in a rigorously defined SR-UC population. Clinical outcomes, namely clinical remission, side effect profile and colectomy rates were meticulously extracted from studies meeting the predefined inclusion criteria. The search scope was delimited to articles composed in English with accessible abstracts, while exclusion criteria comprised other forms of IBD and colitis, non-steroid-refractory UC and steroid-dependent UC. The study also excluded the paediatric population.

### Subgroup analyses

In addition, a sub-analysis of patients exploring the efficacy of oral tacrolimus versus anti-tumour necrosis factor (TNF) therapy in inducing remission in SR-UC. Furthermore, a sub-analysis was conducted to evaluate the utility of topical tacrolimus in cases of steroid-refractory ulcerative proctitis (SR-UP).

### Study selection and quality assessment

The review methodology adhered to the guidelines delineated in the Preferred Reporting Items for Systematic Reviews and Meta-Analyses (PRISMA) statement (see supplementary material).^13^ A panel of three reviewers systematically assessed the quality of all included studies utilising the Oxford Centre for Evidence-Based Medicine 2011 levels of evidence framework, with any discrepancies resolved through consensus.

### Data extraction

Data extraction from the encompassed studies was facilitated by a bespoke template, capturing pertinent details such as authorship, study year, design, baseline population characteristics, treatment regimens, therapeutic outcomes, adverse event profiles and rates of surgical intervention.

### Protocol registration

This review protocol has been prospectively registered with PROSPERO, the international registry for systematic reviews, ensuring transparency and accountability in the research process.

## Results

### Systematic search outcomes

Seventeen studies encompassing 658 patients were identified as meeting the eligibility criteria. The majority of these studies were retrospective (level IV), complemented by two randomised controlled trials and one prospective study.

### The efficacy and safety of tacrolimus in SR-UC

Assessing the efficacy and safety of tacrolimus in SR-UC, 11 studies involving 437 patients were analysed^14–23^ (Table 1). The treatment protocols varied, with oral tacrolimus being the predominant administration route, occasionally supplemented by intravenous (IV) tacrolimus based on individual patient factors. Dosages ranged from 0.1 to 0.5 mg/kg orally and 0.01 to 0.02 mg/kg IV per day, with serum trough levels carefully monitored. The serum trough levels were maintained between 0 and 20 ng/mL at the discretion of the treating physician; in some studies, the trough levels were initially targeted at 10–15 ng/mL for the first couple of weeks, then decreased to 5–10 ng/mL for the remainder of the treatment duration. The continuation of concomitant UC therapies, including corticosteroids and immunomodulators, exhibited variability across studies.

**Table 1.** Studies assessing the efficacy and safety of tacrolimus in SR-UC.

Author	Year	Study design	*n*	Age (years) and gender ratio (%)	Intervention	Serum trough levels	Treatment duration	Clinical response (%)	Remission (%)	Side-effects (%)	Operation (%)
Fellermann et al.^6^	1998	Retrospective	6	–	IV: 0.01–0.02 mg/kg Oral: 0.1–0.2 mg/kg		7 months (0.25–16)	–	66.7 at 9.2 months	–	33.3
Fellermann et al.^14^	2002	Retrospective	38	–	IV: 0.01–0.02 mg/kg Oral: 0.1–0.2 mg/kg for oral		7.6 months	47.3 within 14 days	34.2 at 1 month	Mild: Tremor, hyperglycemia, hypertension and infection	34
Högenauer et al.^15^	2003	Retrospective	9	32 (16–61) 44.5 males/55.5 females	Oral: 0.15 mg/kg/day	10 and 20 ng/mL	15 ± 3 weeks	22.2 at 12 weeks	66.7 at 12 weeks	22.2: severe: thrombocytopenia with intestinal bleeding, bicytopenia. Mild side-effects were common.	11
Yamamoto et al.^16^	2008	Retrospective	27	31 (15–78) 55.6 males/44.4 females	Oral: 0.1 mg/kg body weight/day IV: 0.01 mg/kg body weight/day	0–15 ng/mL to induce remission and 5–10 ng/mL to maintain remission	11 months (1–39 months)	77.8	70.4 in 30 days	18.5 seve: bacterial infection and renal dysfunction	26.9
Ogata et al.^17^	2012	RCT	62	–	Oral: 0.5–1 mg/Twice a day	10–15 ng/mL	2 weeks	50.0 in the tacrolimus group, 13.3 in the placebo group at 2 weeks	9.4 in the tacrolimus group, 0.0 in the placebo group at 2 weeks	Well tolerated with minor side effects	–
Schmidt et al.^18^	2013	Retrospective	130	40 (18–81)	Oral: 0.1 mg/kg	8–12 ng/mL	3 months	–	72 in 3 months	53: mild	14
Landy et al.^19^	2013	Retrospective	25	40.3 (16–75) males♂/females♀	Oral: 0.1 mg/kg/day	5–10 ng/mL	9 months (3.7–18.2)	52 at 6 months	44 at 6 months	44: paraesthesia, tremor, renal impairment	32
Kawakami et al.^20^	2015	Prospective	49	43.8 ± 16.0 51.0 males/49.0 females	Oral: 0.1 mg/kg/day	10–15 ng/mL	4 weeks	89.6 at 4 weeks	75.6 at 4 weeks	35.7: tremors, headache, nausea	6.1
Olmedo Martín et al.^21^	2017	Retrospective	34	37.5 (23.5–44.75) 70.6 males/29.4 females	Oral: 0.15–0.3 mg/kg	5–10 ng/mL	7 months	81 at 12 weeks 81 at 24 weeks	61 at 12 weeks 58 at 24 weeks	53: tremor, renal dysfunction. No severe	20.6
Saifuddin and Harris^22^	2018	Retrospective cohort study using prospectively collated data.	35	36 (18–85) 51 males/49 females	Oral: 0.5 mg/kg	5–20 ng/mL	32 months (6–76)	28.6	14.3 at 32 months (6–76 months)	51.4: renal failure, gastrointestinal. 40 severe| with treatment withdrawal.	62
Hoffmann et al.^23^	2019	Retrospective	22	25.5 ± 5.6 41 males/59 females	Induction: 68.2% of patients started IV: 26 ± 3 μg/kg; 31.8% of patients started oral: 95 ± 31 μg/kg 86.4% continued oral	Induction: 10–15 ng/mL Maintenance: 5–10 ng/mL	128 ± 28.5 days	81.8	36.4 at 705 ± 110 days	45.5: nausea, vomiting, temperature intolerance	31.8

RCT, randomised controlled trial.

Utilising the Lichtiger colitis activity index (CAI) as the primary assessment tool, clinical remission and response rates displayed considerable variability, ranging from 9.4% to 75.6% and 28.6% to 89.6%, respectively. Despite a range of reported adverse events (16.7%–53.0%), predominantly neurotoxic and nephrotoxic in nature, they were generally mild and manageable, although severe reactions (18.5%–40%) – renal failure, thrombocytopenia, intestinal bleeding and bacterial infections – necessitating treatment cessation were noted in select cases. The rate of colectomies among the reviewed studies exhibited significant variance, ranging from 6.1% to 62%, contingent upon the timing and necessity of surgical intervention.

### The efficacy of oral tacrolimus versus anti-TNF in SR-UC

Comparatively, three studies involving 155 patients explored the efficacy of oral tacrolimus versus anti-TNF therapy in inducing remission in SR-UC^24–26^ (see Table 2). Patients in the anti-TNF (infliximab) group were treated with a 5 mg/kg infusion at 0, 2 and 6 weeks, and when clinical response was observed, maintenance treatment of 5 mg/kg every 8 weeks. With tacrolimus, 0.1 mg/kg/day was given with doses adjusted to achieve a target blood concentration of 10–15 ng/mL initially and 5–10 ng/mL thereafter. The main scoring system used to assess disease activity was CAI. No statistically significant disparity in therapeutic efficacy or safety profiles between tacrolimus and anti-TNF agents was discerned. Both treatment modalities demonstrated similar clinical response and remission rates, with colectomy rates exhibiting slight differences.

**Table 2.** The efficacy of oral tacrolimus versus anti-tumour necrosis factor in SR-UC.

Author	Year	Design	*n*	Age (years) and gender ratio (%)	Intervention	Trough levels	Treatment duration	Response (%)	Remission (%)	Side-effects (%)	Operation (%)
Lawrance et al.^24^	2017	Blinded randomised clinical trial	11	48.4 ± 4.9 72 males/27 females	0.5 mg/mL	5.2 ng/L	8 weeks	73	45	30: upper, respiratory tract infection, tremor, headache (Mild)	–
Jaeger et al.^25^	2019	Retrospective study	43	43.6 (16–80) 53.5 males/46.5 females	2 mg of tacrolimus suppository	5.5 ng/mL	76 days	–	60 on day 76	37.2: reversible acute kidney injury, tremor, headache	–
Fehily et al.^26^	2020	Retrospective study	12	31 (22–50) 47 males/53 females	Initial: 1 mg enema, increased to 3 mg over 7 days; maintenance: 1–3 mg used thrice a week	–	20 weeks (3–204)	75	67	25: pruritus or nausea	–

### The use of topical tacrolimus in SR-UP

In the context of SR-UP, 3 studies involving 66 patients evaluated the utility of topical tacrolimus as adjunctive therapy (see Table 3).^27–29^ The treatment method varied from each study, including suppositories (2 mg BD) and enemas (1 mg in 60 mL). The primary endpoint was clinical response by using the Mayo Clinic score and clinical remission by CAI. Despite demonstrating notable therapeutic efficacy, with clinical remission rates ranging from 73.0% to 75.0%, a notable incidence of adverse effects (45.0%–67.0%) was observed, predominantly comprising systemic and local symptoms, albeit generally mild and well tolerated.

**Table 3.** The use of topical tacrolimus in steroid refractory-ulcerative proctitis.

Author	Year	Study design	*n*		Clinical response (%)		Remission (%)		Operation (%)	
			Tacrolimus	Anti-TNF	Tacrolimus	Anti-TNF	Tacrolimus	Anti-TNF	Tacrolimus	Anti-TNF
Endo et al.^27^	2016	Retrospective	47	48	68.1	81.2	53.3	68.8	14.9	8.3
Matsumoto et al.^28^	2017	Retrospective	29	23	58.6	78.2	55.1	56.5	37.9	17.4
Kitayama et al.^29^	2020	Retrospective	79	71	75.9	73.2	25.3	30.1	–	–

TNF, tumour necrosis factor.

## Discussion

The analysis of the studies on tacrolimus therapy for SR-UC reveals varying rates of remission induction. Schmidt et al.^18^ and Kawakami et al.^20^ reported favourable outcomes, with high rates of clinical remission achieved within relatively short treatment durations. In Kawakami et al.’s prospective study, 75.6% of patients achieved remission by week 4, while Schmidt et al.’s retrospective study showed a 72% remission rate within 12 weeks. In both studies, oral tacrolimus was given at an initial dose of 0.1 mg/kg/day, with adjustments made to maintain through whole-blood levels of 10–15 ng/mL. Importantly, patients were permitted to continue receiving their standard immunosuppressive medications.

Contrarily, the findings from Ogata et al.’s^17^ double-blind placebo-controlled trial and Saifuddin and Harris’s^22^ retrospective study paint a less optimistic picture. Ogata et al. reported a significantly lower remission rate of only 9.4% at week 2 of the study, while Saifuddin et al. found that only 14% of patients sustained remission over a median treatment duration of several months (median: 32 months). Notably, in both studies, patients were not on concurrent immunomodulation treatment.

These discrepancies underscore the complexity of tacrolimus therapy and suggest that its effectiveness may vary among different patient populations or treatment protocols. The studies highlight the importance of considering tacrolimus as part of a comprehensive treatment approach rather than as a standalone therapy, in particular, demonstrated that tacrolimus may be more effective when used in conjunction with other immunomodulation therapies. This suggests that combination therapy could potentially optimise treatment outcomes for patients with SR-UC.

The long-term outcomes of tacrolimus therapy remain uncertain due to the limited number of studies in this area. Saifuddin and Harris^22^ and Hoffmann et al.^23^ both reported relatively low rates of sustained remission and a significant proportion of patients requiring colectomy during follow-up periods. Saifuddin et al. conducted a follow-up of their patients for 32 months (range 6–76 months); however, only 14% of the patients who remained on tacrolimus sustained a clinical response. Meanwhile, Hoffmann et al. monitored their patients for 705 ± 110 days after treatment initiation. Among them, 36.4% achieved clinical remission at some point during the treatment. In addition, 32% of the patients underwent colectomy between 5 and 194 days after initiating tacrolimus treatment. This highlights the need for further research to elucidate the role of tacrolimus in long-term maintenance therapy and to identify optimal treatment strategies, potentially involving combination therapy with other immunomodulation treatments.

The rate of adverse events associated with tacrolimus therapy ranged from 16.7% to 53.0%, with neurotoxicity and nephrotoxicity being the most common. In addition, metabolic disorders such as hyperglycaemia and hypomagnesaemia were observed. While most adverse events were mild and manageable, severe reactions necessitating treatment withdrawal were reported, including renal failure, thrombocytopenia, bacterial infections and intestinal bleeding. Consequently, vigilant monitoring for adverse effects is imperative when initiating tacrolimus therapy.

It is worth mentioning the relatively high proportion of patients who would eventually require colectomy despite receiving tacrolimus. The rate of colectomies in the studies assessed in the review varied significantly from 6.1% to 62%. The reasoning behind varied colectomy rates is not clear but could be speculated due to disease severity, varied medication dose, duration of treatment and use of concomitant immunosuppressives.

Anti-TNF-α antibody therapy is an option for the treatment of SR-UC. The comparison between tacrolimus and anti-TNF therapy in inducing remission revealed no significant differences in therapeutic efficacy or safety profiles.^24–26^ In cohorts treated with anti-TNF, clinical response rates ranged from 73.3% to 81.2%, with clinical remission rates spanning from 30.1% to 68.8%. Conversely, in the tacrolimus cohort, clinical response rates ranged from 58.6% to 75.9%, with clinical remission rates between 25.3% and 55.1%. While both treatment modalities demonstrated similar clinical response rates, anti-TNF therapy appeared to offer better long-term outcomes, with a lower rate of colectomy post-treatment initiation. Given the common side effects, the need for parenteral administration and the potential for refractoriness associated with anti-TNF therapy, tacrolimus could represent a viable alternative.

The investigation of topical tacrolimus therapy for SR-UP stems from its potential advantages, notably its targeted delivery to the affected area, minimising systemic adverse effects and obviating the need for multiple immunosuppressive agents in patients. A randomised controlled trial reported a 45% induction of clinical remission, demonstrating superiority over placebo.^27^ Furthermore, two retrospective studies reported clinical remission rates between 60% and 67%, aligning closely with the efficacy of systemic tacrolimus in inducing remission.^28,29^ Of particular note, a retrospective study delved into maintenance treatment, advocating the use of tacrolimus enemas once clinical and endoscopic remission had been achieved. These enemas effectively maintained remission, as confirmed by rectal biopsies indicating mucosal absorption and sustained clinical response and remission.^29^

It’s important to note that while topical tacrolimus exhibited localised therapeutic effects, adverse effects were observed in a significant proportion of patients (45.0%–67.0%). The adverse effects, encompassing systemic manifestations like neurotoxicity and nephrotoxicity, along with local symptoms such as pruritus and peri-anal burning, were generally mild and did not necessitate treatment discontinuation. Interestingly, the studies revealed measurable serum trough levels of approximately 5 ng/mL, within the low therapeutic range of 5–10 ng/mL. This finding is unsurprising given tacrolimus’ efficient absorption through rectal mucosa, bypassing first-pass metabolism in the liver. However, it remains unclear whether these serum trough levels are directly correlated with systemic side effects.^30^

Several factors have hindered the widespread use of topical tacrolimus for the treatment of SR-UP,^30^ including the limited number of studies, variability in delivery methods and dosing regimens, and uncertainties regarding treatment duration. Nonetheless, there exists considerable potential for the expanded utilisation of topical tacrolimus in the management of SR-UP, particularly as a precursor to systemic immunosuppressive therapy. Further research and standardisation of protocols are warranted to optimise its clinical utility. Despite these limitations, topical tacrolimus holds potential as a therapeutic option for SR-UP, particularly as an alternative to systemic immunosuppressants.

## Limitations and strengths

This study provides a comprehensive analysis of tacrolimus therapy for SR-UC and SR-UP, offering valuable insights into its efficacy, safety and potential role in treatment strategies. A key strength of this study is its inclusion of clinical trials and real-world retrospective analyses, which enhances the generalisability of the findings. In addition, the comparison of tacrolimus with anti-TNF therapy provides a balanced perspective on treatment options, aiding clinical decision-making. However, several limitations must be acknowledged. The heterogeneity of the included studies – varying in study design, patient populations, tacrolimus dosing regimens and concomitant therapies – limits the ability to draw definitive conclusions. Moreover, the long-term effectiveness of tacrolimus remains uncertain due to the scarcity of extended follow-up studies, and the high variability in colectomy rates raises questions about patient selection and disease severity. The potential for bias in retrospective studies and the lack of standardisation in topical tacrolimus administration further constrain the applicability of findings. Future research should focus on larger, well-controlled prospective studies to better define optimal dosing, treatment duration and combination strategies for improved patient outcomes.

## Conclusion

Tacrolimus therapy presents a viable treatment option for SR-UC and SR-UP, demonstrating notable efficacy in inducing remission, particularly when used in combination with other immunomodulatory therapies. However, its long-term effectiveness remains uncertain, with variable remission rates, a significant proportion of patients requiring colectomy, and concerns regarding adverse effects such as nephrotoxicity and neurotoxicity. It could be offered as an advantage over other immunosuppressants due to oral medication administration or if patients are intolerant or refractory to their effects. While tacrolimus shows comparable short-term efficacy to anti-TNF therapy, anti-TNF agents appear to provide more favourable long-term outcomes. Topical tacrolimus offers a promising alternative for SR-UP, especially before considering the systemic use of immunosuppressants, though its widespread use is limited by the lack of standardisation in dosing and administration protocols. Given these complexities, further research is needed to refine treatment strategies, optimise dosing regimens and assess long-term safety and efficacy. Ultimately, tacrolimus should be considered as part of an individualised, comprehensive treatment approach tailored to the patient’s disease severity, treatment history and response to prior therapies.

## Supplemental Material

sj-docx-1-taj-10.1177_20406223251333570 – Supplemental material for Systematic review: the use of tacrolimus in steroid-refractory ulcerative colitis Supplemental material, sj-docx-1-taj-10.1177_20406223251333570 for Systematic review: the use of tacrolimus in steroid-refractory ulcerative colitis by Vinay Jangra, Dilsan Yilmaz, Delvene Soares, Fiona Taylor, Stefano Andreani and Alexander Hotouras in Therapeutic Advances in Chronic Disease

sj-docx-2-taj-10.1177_20406223251333570 – Supplemental material for Systematic review: the use of tacrolimus in steroid-refractory ulcerative colitis Supplemental material, sj-docx-2-taj-10.1177_20406223251333570 for Systematic review: the use of tacrolimus in steroid-refractory ulcerative colitis by Vinay Jangra, Dilsan Yilmaz, Delvene Soares, Fiona Taylor, Stefano Andreani and Alexander Hotouras in Therapeutic Advances in Chronic Disease

## Declarations

**Ethics approval and consent to participate:** Not applicable.

**Consent for publication:** Not applicable.

**Availability of data and materials:** Not applicable.
