# Supplementary material for: Systematic review: the use of tacrolimus in steroid-refractory ulcerative colitis
Source: Ther Adv Chronic Dis. 2025 Jun 12;16:20406223251333570. doi: 10.1177/20406223251333570 (PMC12166232; doi:10.1177/20406223251333570)
Supplement: sj-docx-2-taj-10.1177_20406223251333570 – Supplemental material for Systematic review: the use of tacrolimus in steroid-refractory ulcerative colitis [file sj-docx-2-taj-10.1177_20406223251333570.docx]

Records identified through database searching
(n =91)

## Identification

Records after duplicates removed
(n =91)

## Screening

Records excluded
(n = 42, irrelevant)

Records screened
(n = 91)

## Eligibility

Full-text articles excluded
(n = 32)

Full-text articles assessed for eligibility
(n = 49)

## Inclusion

Studies included in qualitative synthesis
(n =17)

Studies included in quantitative synthesis (meta-analysis)
(n =0)
